# Innovative therapeutic targets in chronic sinusitis with nasal polyps^☆^

**DOI:** 10.1016/j.bjorl.2016.03.001

**Published:** 2016-03-29

**Authors:** Claus Bachert

**Affiliations:** a Upper Airways Research Laboratory and ENT Department, Ghent University Hospital, Ghent, Belgium; b Division of ENT Diseases, CLINTEC, Karolinska Institute, University of Stockholm, Stockholm, Sweden

Our current therapeutic options for patients with chronic sinusitis and nasal polyps consist of topical glucocorticosteroids (GCS), carefully indicated short episodes of oral GCS, courses of long term antibiotics (specifically doxyxycline^1^) and sinus surgery. Even with this armamentarium used in combination with pre- and postoperative GCS, studies show high recurrence rates of nasal polyps, with about 80% of regrowth over 12 years. About half of these patients will require surgery again, some of them more than 4 surgeries. There is a clear unmet need for a subgroup of chronic sinusitis patients.

In Europe, the US and certainly Australia, and possibly in other regions, most polyps are characterized by a Th2 inflammatory pattern, with cytokines interleukins (IL)-4, -5, and -13, eosinophils and IgE involved. In fact, this “type 2 inflammation” is associated with severe polyp disease all over the world, and has been demonstrated to predict recurrence of disease and asthma comorbidity, different from eosinophil-poor primarily neutrophilic polyps.^2^ This makes Th2 cytokines and the associated inflammatory cells and their products perfect targets for innovative therapeutic interventions. Very similar mechanisms are also involved in severe asthma and atopic dermatitis, and thus we may hope that treatment approaches that are developed for those diseases will also one day be available for severe nasal polyps.

So far, there have been several proof-of-concept studies performed in nasal polyp disease, all in Ghent, involving the antibodies reslizumab, mepolizumab and omalizumab. These studies have shown that indeed the type 2 inflammation does play a major role in nasal polyps; all three humanized monoclonal anti-bodies targeting IL-5 or IgE have been successful to a certain extent.^3^ Although rather small, the studies demonstrated that polyps shrink over 2–3 months and remain under control for several months thereafter, although treatment approaches were rather limited in time and number of injections. This stimulated hope for further achievements in the future, when nasal polyps would be accepted as targets for registration for major “players” (such as GSK, Sanofi, Novartis etc.), and larger studies would be performed with the aim of achieving registration for the indication of nasal polyps.

This time has now come. On February 2nd, 2016, a first proof-of-concept study with dupilumab, a fully human biologics targeting the IL-4 receptor alpha and interfering with both IL-4 and IL-13 pathways, has been published in JAMA.^4^ IL-4 and IL-13 signal through 2 different receptors that partly overlap in their functions, and they both contain the α subunit of the IL-4 receptor. Via those receptors, IL-4 and IL-13 orchestrate IgE formation, eosinophil recruitment, mucus secretion and many other “typical” events. As both cytokines are prominent representatives of type 2 inflammatory reactions, we performed a double-blind, placebo-controlled randomized study on the effectiveness and safety of dupilumab in patients with chronic sinusitis with nasal polyps with or without concomitant asthma. Polyp scores had to be at least 5 of 8 bilaterally, and polyps had to be refractory to topical GCSs. Sixty patients with bilateral nasal polyposis were included and observed for 4 weeks during topical GCS treatment and then treated either with 300 mg of subcutaneous dupilumab per week (loading dose 600 mg) or placebo for 16 weeks; both groups were receiving daily mometasone furoate nasal spray.

In the verum treated group, the endoscopic nasal polyp score, the CT score according to Lund and Mackay, and each of the typical symptoms as well as nasal flow measurements improved significantly compared to the placebo treated group. Furthermore, the Sino-Nasal Outcome Test (SNOT-22) and the University of Pennsylvania Smell Identification Test revealed clinically and statistically significant effects compared with placebo. More than half of the patients responded with a reduction of the polyp score of at least 2 points, which is the same level of response as 3 weeks of oral application of GCSs. The drug was well tolerated, with the most frequently reported adverse events being naso-pharyngitis, injection-site reactions, and headache (not significant).

Asthmatic patients additionally achieved significantly improved pulmonary function and asthma control test results in comparison with placebo. Concen-trations of biomarkers of eosinophilic inflammation, including serum IgE and chemokines for eosinophils, were also significantly reduced in serum. The results of this study were consistent with previous investigations in patients with severe asthma and atopic dermatitis and confirmed the potential of dupilumab to inhibit TH2-induced inflammation and consecutive symptoms and complaints.

With this study, a new dimension in the management of nasal polyps including comorbid asthma may be revealed. Of course, studies with greater populations and different dosing regimens have to be performed, and registration of the drug may still take two years or more; but for the first time, a registration for the indication of nasal polyps could be achieved. It will then be our task to define the patient groups and the timing of this intervention in the flow of GCS treatment and surgery. Biomarkers might allow us to select patients and predict their response to the treatment, and thus will have impact on our management of the disease. And finally, other biologics will likely come to follow this example.

The future has arrived for innovative treatment of severe nasal polyposis. This implies of course not only new perspectives for us and our patients, but also the need for making us knowledgeable in the immunology of the disease, the intervention and the possible adverse events. We need to become well versed in this area, if we want to maintain the disease within our speciality.

## Conflicts of interest

The author was principal investigator of the studies with biologics for GSK, Novartis and Sanofi-Aventis.
